# Supplementary material for: Are carbon emissions trading and green financial instruments synergistic? -Comprehensive quantitative research based on content analysis
Source: PLoS One. 2024 Mar 7;19(3):e0298601. doi: 10.1371/journal.pone.0298601 (PMC10919601; doi:10.1371/journal.pone.0298601)
Supplement: S1 Data — (ZIP) [file pone.0298601.s001.zip › Data/地级市Z_F工作报告.zip!/╔╜╢1⁄2/╚╒╒╒╩╨/└┤╫╘╣1⁄2╓┌║┼í╛┬φ┐╦╩2╛▌═°í┐_2010.docx]

各位代表：

　　现在，我代表市人民政府向大会作政府工作报告，请予审议，并请市政协各位委员和列席会议的其他同志提出意见。

　　一、2009年政府工作回顾

　　刚刚过去的2009年，是我市经济社会发展面临严峻考验的一年，也是全市人民迎难而上、砥砺奋进，各项工作取得新成就的一年。面对国际金融危机的冲击，我们坚持以科学发展观为统领，紧紧依靠省委、省政府和市委的坚强领导，依靠市人大、市政协的监督支持，依靠全市人民的共同努力，深入贯彻落实中央和省应对国际金融危机、保持经济平稳较快发展的一揽子政策措施，积极作为、科学务实，坚定信心、变中求进，全力保增长、保民生、保稳定，较好地完成了市十六届人大三次会议确定的各项任务，全市经济社会实现平稳较快发展，主要经济指标增幅在全省保持前列。初步测算，全年实现生产总值860亿元，比上年增长14.5%，三次产业比例由上年的10.7∶54.3∶35调整为10∶55.8∶34.2。实现地方财政收入43.48亿元，增长19.68%。完成规模以上工业增加值450亿元，增长30%。完成规模以上固定资产投资630亿元，增长23%。进出口总值稳居全省第四位，达到83.5亿美元。城镇居民人均可支配收入15800元，农民人均纯收入6550元，分别增长9.6%和8.5%。

　　(一)应对国际金融危机、扩内需保增长取得阶段性成果。把保持经济平稳较快增长作为经济工作的首要任务，抢抓国家实施积极财政政策和适度宽松货币政策的重大机遇，全力以赴扩内需、保增长。深入开展“大项目建设年”活动，确定了境内总投资2686亿元的138项重大建设项目，开工建设86项，完成投资208.76亿元。集中力量争取国家和省扩大内需项目与资金，共争取中央新增预算内投资项目143个，争取中央投资3.37亿元、省调控资金9.68亿元、地方政府债券2.1亿元，我市投入配套资金15.35亿元。财税收入保持平稳较快增长，税收占地方财政收入的比重达到86.35%。金融对保增长发挥了重要支撑作用，市政府分别与工商银行、农业银行、中国银行、建设银行、招商银行签署了战略合作协议，获得三年授信860亿元；金融机构存贷款余额分别较年初增加198.87亿元和255.71亿元，新增贷款是上年的5.5倍。资本市场融资取得新成效，日照港非公开发行股票募集资金12.8亿元，累计上市融资已达32亿元。恒宝食品进入境外上市程序，一批上市后备资源初步形成梯次结构。面对国内外复杂的经济形势，坚持招商引资的力度不减、目标不变，出台了鼓励外商投资优惠政策和招商引资引荐人奖励政策，组织举办了海内外知名企业家齐鲁行、东部沿海国际经贸论坛、物流产业论坛、蓝色产业科技论坛和赴欧美、日韩、香港、深圳、陕西、甘肃、长三角等一系列招商推介活动，共招引和推进千万元以上的项目985个，实际到位资金268.9亿元，实际到账外资3.6亿美元。坚持把保企业作为保增长的关键环节，制定出台了多方面共计181条政策措施，全方位为企业“加油松绑减压”。在财力紧张的情况下，安排1亿元设立工业发展专项资金，有效提升了企业应对危机的能力。全市规模以上工业企业预计实现主营业务收入1800亿元、利税160亿元、利润110亿元，分别增长20%、15%和10%。港口运输在危机中实现了逆势上扬，全年完成货物吞吐量1.81亿吨，增长20.1%，增幅居全国沿海十大港口之首，吞吐量比2005年翻了一番多，相当于四年再造了一个日照港。出台了促进消费结构优化升级和鼓励房地产、文化旅游产业发展等多方面的措施，认真落实家电、汽车、摩托车下乡和家电以旧换新政策，努力挖掘城乡消费潜力。全市预计实现社会消费品零售总额252亿元，增长19%。在着力扩大内需的同时，千方百计稳定外需，出台了支持外经贸企业发展的政策措施，全市进出口形势总体向好；实现对外承包劳务营业额1.1亿美元，外派劳务8000人次，完成境外投资3714万美元，分别增长20%、17.5%和26.3%。在遭受国际金融危机冲击的严峻形势下，我们迅速扭转了经济增速下滑的态势，全市经济呈现出企稳向好的局面，保持了社会和谐安定。

　　(二)产业结构得到进一步优化。充分利用金融危机带来的“倒逼机制”和“洗牌机制”，不失时机地推进经济结构调整，加快构建现代产业体系。精心编制蓝色经济区发展总体规划和鲁南临港产业区规划，立足于建设“我国东部沿海重要的临海产业基地、区域性国际航运物流中心、滨海文化和旅游名城、海洋科技教育创新区”的战略定位，确立了“一带三轴八区十大重点产业”的规划格局。经过积极争取，石油化工基地和海洋装备制造基地被纳入省蓝色经济区规划。制定并启动了钢铁、汽车、石化、轻工、纺织、装备制造、船舶、电子信息八大工业调整振兴规划，计划三年实施137个调整振兴项目，年内已开工建设81个，完成投资131亿元，现代威亚发动机二工厂和模具工厂、青岛啤酒30万千升迁扩建、石大科技一期、锦湖金马胶乳等一批新项目建成投产，全市新增主营业务收入过亿元的企业70家，利税、利润过千万元的企业119家，规模以上工业企业累计达到1000家。日照钢铁精品基地被国务院和省政府列入钢铁产业调整振兴规划，山钢与日钢正式签订了资产重组合作协议，资产重组及其他前期工作正在着力推进。科技创新稳步推进，清大华创科技孵化器和首都师大动漫基地投入运营，五征企业技术中心被列为国家级企业技术中心，全市21个产品被评为山东名牌产品，预计全年实现高新技术产值330亿元，增长33%，增幅居全省首位，占规模以上工业产值的比重提高2个百分点。服务业和特色经济蓬勃发展，预计全年实现服务业增加值294亿元，增长10%，我市被省政府表彰为“服务业发展先进市”。港口物流业进一步向中原地区及内陆腹地推进。组织举办了国际海滩旅游文化节、啤酒节、绿茶节、艺博会等宣传促销活动，进一步提升了日照的知名度。全年共接待国内外游客1741万人次，实现旅游总收入100亿元，分别增长18.8%和21.9%。电子商务产业异军突起，龙鼎电子年交易额达到3620亿元，和易电子日平均交易额突破1亿元。服务业重点园区、重点企业、重点市场建设迈出新步伐，40个载体项目年内新增投资103亿元。在加快发展新兴产业的同时，加大落后产能淘汰力度，下决心推进节能减排，开展了集中爆破拆除落后产能行动，共淘汰立窑水泥生产线14条，拆除燃煤锅炉4台；完成既有建筑节能改造45万平方米。积极开展“整治违法排污企业、保障群众健康”环保专项行动，建成污水和垃圾处理工程11处，在全省污染物减排和环境质量改善考核中我市获得三个一等奖。在第二届中国绿色发展高层国际论坛上，我市荣获“中国十佳绿色城市”称号；在中国创业城市高层论坛上，我市荣膺首批“中国创业之城”。

　　(三)社会主义新农村建设迈出新步伐。大力开展粮食高产示范创建活动，粮食总产连续五年突破100万吨，达到112.42万吨，创2001年以来新高；单产达到419.6公斤，创1997年以来新高。新增畜禽规模养殖场45个，新发展贝藻参浅海立体养殖1万亩，争创省级以上水产健康养殖示范场4家，增殖放流水产苗种2.4亿尾粒。肉蛋奶总产量增长9.7%，水产品总产量达到47.86万吨。启动了三年造林绿化行动，完成造林面积9.55万亩，是2004年以来造林面积最多的一年，森林覆盖率提高到35.2%。新发展茶园2.2万亩，是近十年来发展最快的一年，岚山区被确定为省级优质绿茶生产基地。新建烟叶生态村65个，植烟面积达到8.8万亩。农业防灾减灾体系进一步完善，美国白蛾等农业病虫害防治、动物疫情防控、森林防火等工作扎实有效。农业产业化、农民组织化程度进一步提高，规模以上农业龙头企业发展到269家、农民专业合作社达到1098个，年内分别新增28家和871个。农村土地承包经营权流转稳妥有序推进，集体林权制度改革试点顺利启动，水管和兽医管理改革逐步深化。大力加强农村基础设施建设，实施了沭河莒县段综合治理二期工程和小仕阳、长城岭、石亩子、巨峰4座大中型水库除险加固工程，完成小型水库除险加固120座，治理水土流失面积60平方公里，改造中低产田4万亩，建设旱作农业核心示范区2万亩，发展节水灌溉4.3万亩。建成了220千伏招贤、110千伏碑廓、虎山等输变电工程。新建农村硬化路315公里，成为全省第一个实现全部行政村通硬化路的市。认真落实强农惠农政策，发放粮食直补等各类补贴2.87亿元，推广农户科学储粮示范仓2.1万个。编制了村镇体系建设规划，启动实施了农村住房建设与危房改造工程，与中国建材集团联合开展了农村新型住宅建设产业化试点。新建农村住房3.3万户，改造农村危房1945户。新建改造农村自来水工程162处，农村自来水普及率提高到90%。组织实施了农村中小学校舍维修改造、教学仪器更新、“211”建设等基础性工程，补充中小学教师640名，提前一年完成了农村义务教育债务化解。改造乡镇卫生院业务用房11处，建设村卫生室业务用房547处、配备医疗设备563处。各乡镇、街道普遍建成了综合文化站，70%的行政村建成了文化大院，建设农家书屋800家。累计建成农村社区服务中心73处、200平方米以上的乡村综合超市279处，县乡村三级商品连锁配送网络覆盖率达到88%。全市广大农民的物质文化生活条件不断改善，精神风貌发生着新的变化，欣欣向荣的社会主义新农村景象正在日益展现！

　　(四)港路基础设施建设取得重大突破。山西中南部至日照港铁路大通道开工建设。青日连铁路已确定规划，即将动工兴建。新菏兖日铁路电气化改造以及日照港疏港高速、长深高速公路日照段建设顺利推进。335省道三庄至莒县段建成通车。日照港参与投资的东(都)平(邑)铁路、枣(庄)临(沂)铁路建设进展顺利。日照保税物流中心(B型)正式封关运营，鲁南国际贸易与航运服务中心口岸通关部分投入使用。与中石油合作的日照至东明输油管道开工建设，与中石化合作的岚山港区30万吨级原油码头及与之配套的日照至仪征输油管道已经国家发改委核准，为打造国内重要的石化基地奠定了坚实基础。投资22.6亿元，新建续建了石臼港区西区三期、矿石二期等13项港口重点工程，新增生产性泊位9个。日照水库向市区供水三期工程提前完工，新增日供水能力10万吨。同时，完成了全市土地利用总体规划修编并通过评审验收，在全省率先编制完成了农村建设用地挖潜利用规划，为建设发展创建了更为合理的空间环境。这一系列基础设施重大项目的突破，将为我市连接东西、辐射内陆、贯通南北、融合沿海创造优越的条件，为今后的长远发展积蓄后劲、打牢基础，必将强有力地提升我市在竞相发展中的综合竞争优势，为建设海洋特色新兴城市提供坚实的基础支撑！

　　(五)城市规划建设管理水平进一步提高。开展了中心城区设计国际咨询，规划初稿已编制完成。高起点编制了日照北部地区概念规划、市区基本生态线控制规划和钢铁精品基地规划。阳光海岸带南段整治工程进展顺利，“梦幻海滩”特色初步显现。游泳馆主体工程已经完工。天然气长输管道建成通气。12个城中村改造全部启动，累计拆迁房屋39万平方米，建设安置楼40.4万平方米，回迁居民1610户。完成了天津路等8条道路建设和黄海一路等3条道路改造，新增城市绿地275万平方米。对口援建桃龙乡驻地七项工程全部交付使用，援建北川新县城项目扎实推进。牢固树立和谐城管理念，加强规划执法，强化市容监管，城市管理水平进一步提升。城市社区建设扎实推进，有1个街道、2个社区被命名为全国和谐社区建设示范单位。城市环境综合整治定量考核继续保持全省第一，被省政府表彰为“生态省建设先进市”。在第十一届中国住交会上，我市荣获“中国城市科学发展创新奖”。日照水上运动基地荣获“新中国成立60周年百项经典暨精品工程”称号。特别是经过全市人民多年坚持不懈的努力，日照一举获得2009年度“联合国人居奖”，这是我市坚持“四大战略”取得的重大成果，是全市人民的骄傲和自豪！

　　(六)保障和改善民生取得新成效。在财政收支压力加大的情况下，市财政统筹用于改善民生的资金达41.26亿元，比上年增长29.87%。12件为民办实事项目圆满完成。在全省率先开展了企业公开承诺不裁员活动，采取降低社保费率、提供稳岗补贴等措施稳定就业，全年新增城镇就业4.37万人，有组织转移农村劳动力2.55万人，城镇登记失业率控制在2.85%。全面推进城镇居民基本医疗保险，参保率达到95%，城镇基本医疗保险覆盖率提前两年实现了国家确定的90%的目标。积极开展城镇职工社会保险扩面征缴，实施新型农村社会养老保险试点，新增城镇职工社会保险参保人员5.2万人，企业退休人员养老金提高13%，城市低保标准提高到每月276元，农村低保标准提高到每年1200元。建立动态调整、物价与生活补贴联动、分类施救、准入退出、“三公示长监督”五项新型社会救助机制的做法在全省推广。精心组织实施“五有惠残共享工程”，市社会福利中心开工建设。经济适用房货币直补户数达到1264户，为194户低保或特困家庭发放了廉租房补贴，在市区配建廉租房200套，首批22户已经入住。扎实推进农产品、食品、药品“三品”安全体系建设，“放心肉”工程列入全国试点，“放心早餐工程”启动，粮食质量检测中心投入使用。向全市所有家庭免费发放了《市民应急避险知识手册》和《民生手册》2009年增编版。大力加强应急管理体系建设，组建了市应急救援支队和专业救援分队。深入开展“安全生产责任落实年”活动，生产安全事故起数下降19.6%，其中道路交通事故下降17.3%，火灾事故下降32.2%。积极构筑“五网”防控体系与“合成化”现代警务体系，社会治安群众满意度居全省第一，我市被中央综治委表彰为“全国社会治安综合治理优秀市”。

　　(七)精神文明、社会事业、民主法制、双拥共建等工作扎实有效。成功举办了庆祝新中国成立60周年、地级日照市设立20周年系列活动，极大地增强了全市人民的爱国爱市热情，营造了凝心聚力、干事创业的浓厚氛围。以“迎全运盛会、创文明城市”为主题，广泛开展“四心”教育、生态文明村镇创建和未成年人思想道德教育，城乡文明水平进一步提高。成功承办了第十一届全运会日照赛区比赛，被省委、省政府表彰为“最佳赛区”。我市运动员在全运会上勇夺金牌3.5枚，实现了竞技体育的历史性突破。成功举办了2009中日韩国际帆船赛、首届全市老年人运动会。翟墨驾驶“日照”号单人无动力帆船环球航海胜利返航。大力加强公共文化设施建设，市文化艺术博览中心和莒州博物馆建成启用，市级综合文化中心暨日照图书馆奠基开工。新编大型历史吕剧《毋忘在莒》赴省晋京演出获得圆满成功。对市区10万户居民实施了数字电视整体转换。医药卫生体制改革全面启动，新型农村合作医疗参合率达到97.39%。深入开展环境卫生“消杀灭”活动，稳步推进免疫规划，甲型H1N1流感和手足口病等重点传染病防控工作扎实有效。通过实行强弱学校联合办学、新建优质学校、改革招生制度等措施，积极推动市区义务教育均衡发展。职业教育得到进一步发展和加强。综合治理人口问题取得明显成效，人口自然增长率控制在3.67‰，合法生育率达到97.39%。国防动员、民兵预备役、优抚安置和双拥共建工作得到加强，全民普法与依法治市规划深入实施。

　　(八)政府自身建设进一步加强。按照中央和省委、市委的统一部署，扎实开展深入学习实践科学发展观活动，政府各级干部的科学发展理念更加强化。出台了《关于进一步转变机关作风，提高行政效能，推动政府工作全面提速的意见》，对改善政务服务作出21条规定，投资软环境进一步改善。建成了网上审批和电子监察平台，实现了所有行政许可审批事项和便民服务事项网上办理，审批件提前办结率达71.4%。自觉坚持依法行政，主动接受市人大及其常委会的法律监督和市政协的民主监督，全年共办理人大代表建议55件、政协委员提案199件。行政监察和审计监督进一步加强。新开展了市领导与市民网上对话，民意诉求渠道更加畅通，解决了一批事关群众切身利益和社会关注的问题。全面落实党风廉政建设责任制，认真贯彻中央《关于党政机关厉行节约若干问题的通知》要求，全市机关事业单位出国经费、车辆购置运行费在近三年平均数基础上分别下降20%和15%。

　　各位代表：

　　过去的一年是我市发展史上极不平凡的一年，在前所未有的困难与重大历史机遇交织的复杂形势下，我们有效应对了国际金融危机的严峻挑战，保增长、保民生、保稳定的目标胜利实现。我们成功争取了一批事关日照长远发展的重大产业和基础设施项目，特别是以日照钢铁精品基地为重点的鲁南临港产业带被省委、省政府确定为山东半岛蓝色经济区重点打造的“一区三带”之一，使日照区域性枢纽、新增长极的作用进一步凸显，在全省发展大格局中的战略地位空前提高，一座优势突出、独具魅力的海洋特色新兴城市蓄势待发，展现出更加广阔而又清晰的发展前景！

　　过去一年的成绩来之不易，形势的好转远远超出年初的预期。这是在党中央、国务院、省委、省政府和市委的坚强领导下，全市上下团结一心、艰苦奋斗的结果。面对国际金融危机冲击，市委、市政府及时引导全市各级科学判断形势，认清机遇与挑战，坚定必胜信心；按照“出手要快，出拳要重，措施要准，工作要实”的要求，不失时机地向国家和省争取项目、争取资金、争取政策，迅速出台保企业等一揽子政策措施，实现了“危”中求“机”、化“危”为“机”；结合开展深入学习实践科学发展观活动，继续参与和推动“向南看、向北看”解放思想大讨论，组织学习先进地区应对危机促发展的成功经验，进一步解放了思想、提升了境界。一年来我们在应对国际金融危机中所积累的实践经验，对日照未来发展来说必将成为一笔宝贵财富，而在这场危机中所展现出的全市人民战胜危机、克服困难的信心、勇气和高尚情操更会成为日照未来发展的强大精神动力和物质力量！在此，我代表市人民政府，向辛勤工作在全市各行各业的广大劳动者和建设者，向给予我们监督支持的各级人大代表、政协委员，向各民主党派、工商联、无党派人士、各人民团体，向离退休老领导、老同志，向驻日照人民解放军和武警官兵，向所有关心支持日照建设发展的各界人士和海内外朋友，表示崇高的敬意和衷心的感谢！

　　在充分肯定成绩的同时，也应当清醒地看到，国际金融危机的影响依然存在，国际市场继续低迷，经济全面企稳回升的基础还不稳固，经济增长的内在动力依然不足，保持经济平稳较快发展的任务仍很艰巨；我市经济总量小、人均占有低与产业结构层次不高的矛盾并存，主导产业不大不强，装备制造业、现代服务业、高新技术产业发展滞后，节能减排任务重、压力大；城乡发展不够协调，县域经济基础薄弱；基层公共服务设施有待完善，部分群众在就业、就医、教育、社保、住房等方面还存在困难；政府个别部门和单位服务意识不强，办事效率不高，经济发展环境还需进一步改善；个别工作人员为政不廉的现象依然存在。对这些困难、矛盾和问题，我们一定高度重视，增强忧患意识，采取更加有力的措施，认真加以解决。

　　二、2010年政府工作任务

　　今年是全面完成“十一五”发展规划的最后一年，也是应对国际金融危机的关键之年，做好今年的政府工作，对于夺取应对国际金融危机冲击的全面胜利、保持经济平稳较快发展、保障和改善民生、确保“十二五”规划顺利启动实施，具有十分重要的意义。综合分析今年的形势，我们面临着稳增长与调结构的双重压力，挑战与机遇并存，机遇大于挑战。从外部环境看，世界经济已出现复苏迹象，外部需求有望逐步恢复，但影响经济发展的不确定因素仍然较多，扩大外需的压力仍然很大。在这样的大环境下，中央确定继续实施积极的财政政策和适度宽松的货币政策，保持宏观经济政策的连续性和稳定性，提高宏观经济政策的针对性和灵活性，为保持经济平稳较快发展提供了基本保证。省委、省政府倾力打造山东半岛蓝色经济区，全力争取将其纳入国家“十二五”规划，上升为国家战略，把以日照钢铁精品基地为重点的鲁南临港产业带列为重点打造的“一区三带”之一，将日照作为鲁南经济带的龙头来培育，在重大生产力布局和基础设施建设方面给予倾斜，为我市科学发展、跨越发展、和谐发展创造了千载难逢的历史机遇。全市广大干部群众战胜危机、克服困难的信心更足，谋发展、干事业的愿望更强，心往一处想，劲往一处使，使新一年的建设发展有了强有力的支撑。只要我们紧紧依靠省委、省政府和市委的坚强领导，坚定不移地贯彻落实科学发展观，以更加坚定的信心、更加开阔的视野、更加务实的作风，抓住新机遇，增创新优势，实现新跨越，就一定能够谱写出日照科学发展的壮美篇章！

　　今年政府工作的总体要求是：认真贯彻党的十七大、十七届三中、四中全会和中央、全省经济工作会议精神，以科学发展观总揽全局，紧紧围绕打造蓝色经济区、发展鲁南临港产业区、建设海洋特色新兴城市，深入实施“四大战略”、“双轮驱动”，加快构建现代产业、民生保障、公共安全、生态环境“四大体系”，按照“五个更加注重”的要求，坚持积极作为、科学务实、坚定信心、变中求进，坚持在发展中求转变、在调整中促发展，着力保发展、转方式、调结构、惠民生，着力发展蓝色经济和战略性新兴产业，着力增强经济社会发展的可持续性、协调性、创新性，努力开创日照科学发展、跨越发展、和谐发展新局面。

　　综合考虑各方面因素，今年经济社会发展的主要预期目标是：生产总值增长13%，地方财政收入增长13%，规模以上工业增加值增长20%，规模以上固定资产投资增长23%，社会消费品零售总额增长17%，进出口总值增长10%，城镇居民人均可支配收入和农民人均纯收入分别增长9%和8%。主要约束性目标是：万元GDP能耗、二氧化硫、化学需氧量排放完成省政府下达的任务目标，人口自然增长率控制在6‰以内。

　　各位代表：

　　转方式、调结构将是今年经济工作的一项重大任务和主攻方向。对我市来说，由于经济基础薄弱，实力不强，应该重点通过增量的优化和质量水平的提高带动整个经济结构的调整，促进发展方式的转变，并以此推动落后产能的淘汰，从而把握增量与存量双调整的协调统一，实现经济总量、总体实力和质量效益的全面提升。在经济发展的推动力上，针对日照的实际，今年应该着重于在稳定外需、扩大内需的同时，充分发挥投资的拉动作用，以投资结构的优化促进经济结构的调整。

　　今年应当重点做好八个方面的工作：

　　(一)努力保持经济平稳较快发展。充分利用国家继续实施积极财政政策和适度宽松货币政策的有利时机，努力扩投资、促消费、稳外需，确保经济平稳较快增长。

　　一是保持投资的强劲增势。继续组织实施“大项目建设年”活动，对大项目建设从供地、审批等环节给予全力支持，促其快投资、快建设、快见效，年内重点组织实施169个重大项目，估算总投资2794亿元，年内确保完成275.8亿元。继续加大对上争引力度，再筛选一批符合国家产业政策、对结构调整和产业升级具有引领作用的好项目，争取更多地列入中央和省支持范围。综合运用产业、土地、金融、环保、资源利用等政策，引导社会资金投向交通、能源、农林水利、社会民生、高新技术、技术改造、节能环保、现代服务业等重点领域和薄弱环节。切实抓好中央扩内需项目的管理和实施，集中保续建、保收尾，防止出现“半拉子”工程。扩大市场准入范围，进一步激活社会投资。

　　二是强化对企业的支持保障。继续贯彻落实并完善好扶持企业发展的一揽子政策措施，继续安排并利用好专项资金，支持引导企业在结构调整、自主创新、现代服务业发展、节能减排等方面发挥主体作用。继续严格控制涉及企业的检查、评比、达标、表彰活动和各种收费，确保各类检查收费不反弹，并努力减轻企业负担。认真贯彻落实《做大做强金融业的意见》，深入推进银企合作，优化信贷结构，扩大信贷投放，特别是加大对中小企业的信贷支持力度。加强地方金融体系建设，进一步搞好小额贷款公司、村镇银行发展等试点工作。落实好鼓励企业上市的优惠政策，引导更多的企业上市融资。

　　三是进一步扩大消费。采取简化手续、完善网络等措施，将国家出台的增加家电下乡品种、扩大补贴范围，延长家电以旧换新、汽车下乡政策时限等政策落到实处。积极引导和促进汽车、住房等大宗商品消费。加快发展“放心早餐工程”和社区商业、物业、家政等便民消费，扩大文化娱乐、体育健身、旅游休闲消费。深入推进“万村千乡”和“下乡产品流通网络”工程，支持农家店和农村商品配送中心、销售网点、售后服务网点建设。鼓励企业研发新产品，完善产品的外观、功能和特性，主动引领消费。切实增强我市的对外吸引力，扩大消费群体和消费领域。努力开拓渠道增加城乡居民特别是中低收入群体收入，进一步完善最低工资制度，健全社会保障体系。积极倡导和引导消费，使预期消费合理地转化为即期消费。

　　四是千方百计在稳定外需的基础上扩大出口。深入实施市场多元化战略，支持企业通过参加境外展会、建立境外自主营销网络等措施，深度发掘美欧日韩等重点市场，大力拓展东盟、南美、非洲等新兴市场。积极调整优化出口产品结构，抓好科技兴贸出口基地和出口农产品质量示范区建设，推动加工贸易转型升级，进一步提高我市出口产品的市场竞争力。加快实施“走出去”战略，重点推动兴业集团、华信工贸、中瑞物产等企业在菲律宾、墨西哥等国家的矿产资源开发项目。积极开展境外工程承包，带动劳务出口。

　　五是切实抓好财政增收节支。坚持把稳增长、调结构与加强财源建设结合起来，积极落实财源建设奖励政策，加大考核扶持力度，不断培育壮大新兴产业，大力培植优质高效财源，努力发展财源经济。加快推进企业主辅分离，完善国有资源有偿使用制度，扩大地方财政收入来源。依法加强税收征管，完善税源管控体系，做到应收尽收。大力优化支出结构，坚持突出重点，有保有压，减少公用经费和一般性开支，把有限的钱花在刀刃上。

　　(二)加快启动鲁南临港产业区规划建设。牢牢把握鲁南临港产业带被省委、省政府列入蓝色经济“一区三带”之一的重大历史机遇，按照海陆统筹的思路，加快推进“港城、港带、港桥、海陆”一体化，大力开发海洋经济、海洋产业、海洋科技、海洋文化、海洋资源，保护海洋环境，努力在临港产业区建设上开好头、起好步。

　　一是坚持以高水平的规划为先导。积极对接山东半岛蓝色经济区总体规划，进一步调整完善港口交通运输专项规划、钢铁精品基地配套产业发展规划、基础设施建设专项规划和区域建设用海规划，更好地适应鲁南临港产业带建设需要。健全完善园区和产业基地建设规划，争取更多的示范园区、基地和重点项目列入省规划。积极推动青岛—日照—潍坊区域协调发展规划编制工作，搞好日照国际海洋城各项前期工作，及时启动规划建设。

　　二是加快推进重大项目建设。围绕钢铁及配套产业、石油化工、汽车及海洋装备制造等十大重点产业，加快引进龙头项目，拉长产业链，实现集群式发展。特别是要把钢铁精品基地规划建设作为头号工程，加快推进山钢与日钢资产重组，确保新公司按时挂牌运营，租赁经营期平稳过渡。进一步做好基地选址、建设用地、拆迁安置、港口配套、水电保障等前期工作，为项目建设创造条件。全力配合省直有关部门做好钢铁精品基地报批工作，力争早获批、早开工。统筹搞好配套产业区、商务区、生活区、基础设施工程等规划建设，发挥钢铁精品基地对全市经济发展、财政增收、社会就业、城镇化进程等综合带动作用。

　　三是强化基础设施载体支撑。新建续建岚山港区30万吨级原油码头、南作业区防波堤等10项港口建设重点工程。开工建设青日连铁路、坪岚铁路扩能改造、日照至仪征输油管道及配套工程，加快建设山西中南部至日照港铁路大通道、日照至东明输油管道、新菏兖日铁路电气化改造以及日照港疏港高速、长深高速日照段、342省道汾水至临沂段拓宽改造等工程，积极推动日潍、岚临高速公路项目，适时启动204国道城区改线项目。加快推进“西水东调”前期工作和马陵水库、户部岭水库向市区供水工程规划建设，构建城乡一体的水资源安全保障体系。加快城乡电网建设，年内投资6.4亿元，建设稍坡、秦楼220千伏和汪家台、南湖110千伏等输变电工程。

　　(三)更加积极主动地转方式、调结构。继续按照增量与存量“双调整”，以“增量”调整为主的原则，致力于打造蓝色经济，依托海洋推动海陆经济一体化发展；打造绿色经济，坚持环保优先；打造特色经济，形成日照发展的新优势。

　　一是深入推进工业调整振兴。进一步落实好八大工业调整振兴规划，继续组织实施137个工业调整振兴项目，滚动实施百项重点工业项目，重点推进现代威亚三期、森博浆纸二期和三期、森达美棕榈油、华海造船二期、锦湖金马化工二期、轻卡和微型车、晨曦重油制烯烃、黄海粮油二期等投资过亿元的大项目建设。组织实施战略性新兴产业培育计划，积极引进高新技术产业和行业龙头企业，重点培育新能源、新材料、新医药、新信息等新兴产业，年内完成高新技术产业产值380亿元。加快技术改造和设备更新，鼓励企业嫁接高新技术，提高工艺装备水平和产品附加值。加强科技创新体系建设，注重培养引进高层次人才，支持企业与高等院校、科研单位合作共建技术中心，年内建成市级以上工程技术研究中心和企业技术中心30家。

　　二是加快发展服务业。大力发展港口物流业，积极在腹地城市特别是沿新亚欧大陆桥中西部地区建立港口物流区域枢纽或设立“无水港”，全年港口货物吞吐量突破2亿吨、集装箱运量突破100万标箱。全面落实物流业振兴规划，加快中瑞国际物流中心、中盛国际商务港等重点园区和骨干企业建设，发展壮大第三方物流。发挥好保税物流中心“境内关外”的政策优势，吸引大型物流企业入驻，并逐步向保税港区的方向发展。充分发挥鲁南国际贸易与航运服务中心的作用，推进“大通关”和电子口岸建设。进一步发展金融、保险、证券业。坚持打造四季游、休闲度假游和高档精品游，推进重点旅游项目和岚桥、开元等高档休闲度假酒店建设，积极开拓客源市场，年内旅游人数力争突破2000万人次、旅游收入达到120亿元。进一步做大做强电子商务企业，积极争创“中国电子商务示范城”。逐步增加服务业引导资金，支持服务业“三大载体”建设。

　　三是坚持不懈地抓好节能减排。坚持增量、存量“双调整”的思路，立足于打造循环经济、绿色经济、低碳经济，加快发展先进制造业、现代服务业和高新技术产业，在做大做优“分母”上下功夫。严格落实节能减排目标责任制，继续推进能效对标活动，抓好节能技术改造，强化对重点区域、重点行业和重点企业能耗、水耗、污染物排放控制，年内再淘汰一批落后产能，实现主要产品能耗、重点工序能耗持续下降。加大工程减排、结构减排和管理减排力度，进一步完善污水处理厂及配套设施，确保稳定达标排放。

　　(四)高度重视“三农”工作和城镇化建设。坚持稳粮保供给、增收惠民生、改革促统筹、强基增后劲，推动“三农”工作再上新水平。

　　一是大力发展现代农业。坚守基本农田、耕地面积、粮食总产“三条底线”，实施高产创建示范工程，大力推广良种良法配套栽培技术，年内新发展节水灌溉面积4.5万亩，改造中低产田16.3万亩，确保粮食总产稳定在100万吨左右。坚持产业化、特色化、标准化方向，积极开展“农业结构调整年”活动，大力发展茶叶、瓜菜、林果、水产、畜牧等高效生态农业和特色农业，推进无公害产品、绿色食品、有机食品认证。积极培育壮大农业龙头企业，大力发展农民专业合作社。全面落实强农惠农政策，确保各项补贴不折不扣地落实到农民手中。

　　二是强化农村基础设施建设。抓好小仕阳等4座大中型水库、89座小型病险水库、36座塘坝除险加固及潮白河东港段综合治理等工程建设，年内基本完成病险水库除险加固。抓好青峰岭水库灌区节水改造二期和莒县小型农田水利基本建设重点县工程，新解决95个村庄、7.1万人的农村饮水安全问题。加快推进农村住房建设与危房改造，积极推进农村新型材料住宅产业化，同步实施供排水、照明、污水垃圾处理等配套设施建设，年内力争新建农房4万户，改造危房1.4万户，推广应用农村新型住宅800户。进一步提高农村公路通达能力，着力提升中心镇、中心村周边公路等级，推动“村村通”向“网络化”、“村内通”发展。稳步推进农村社区服务中心建设，新建社区服务中心10处。

　　三是加快新型城镇化进程和土地集约节约利用。把新型城镇化作为扩大内需、调整结构、保持经济平稳较快发展的重要抓手，按照循序渐进、节约土地、集约发展、合理布局的原则，着力提升日照中心城区、县级城区和中心镇的带动能力。把小城镇作为统筹城乡发展的重要节点，探索对国家级、省级中心镇实施扩权强镇，稳妥开展撤乡并镇，大力推动各具特色的小城镇建设。坚持以产业为支撑，以基础服务设施为保障，以政策为导向，以放宽城镇户籍限制为条件，引导城镇人口规模与结构合理增长和优化，并使在城镇稳定就业和居住的农民特别是新生代农民工有序转为城镇居民。引导各区县着力打造具有自身特色、三次产业结构合理的县域经济。编制县乡两级土地利用总体规划，推进土地开发整理，搞好建设用地挖潜，保持耕地总量动态平衡，并协调好农村建设用地减少和城市建设用地增加的关系，搞好增减挂钩试点。

　　(五)以更大的力度推进招商选资和体制创新。坚持以我为主、择优选资，围绕重点打造的十大产业，大力开展产业招商、专业招商、园区招商和企业招商，进一步扩大和深化与现代起亚汽车集团、新加坡金鹰集团、中石化、中石油等国字号、外字号、民字号大企业大集团的战略合作，争取更多的大项目、高科技项目、新兴产业项目、外向型项目和“二三期项目”落户日照。组织好已签约项目的实施，着力推进山东重工重卡，韩国、香港汽车制造及其发动机、零部件，昊燃、国电和大唐风电等新兴能源，天一生物科技，中新建材，紫光科技等一批新兴产业项目，加快培育新的经济增长点。认真落实鼓励外商投资优惠政策和招商引资项目引荐人奖励政策，组织开展赴新加坡、日韩、欧美、香港和上海、西安、青岛的招商推介活动，为招商选资搭建平台。继续加大接轨青岛力度，积极推动与鲁南各城市共建临港产业带。

　　进一步解放思想，大胆探索，着力推进重点领域、关键环节的改革。积极争取鲁南临港产业区综合配套改革试点方案早日获批，在实际工作中不等不靠，积极进行在开放、合作、财税、金融、土地、科技、资源环境等方面的体制机制创新，努力建成对外开放的先行区、吸引外资的密集区、高新产业的集聚区、生态文明的示范区。对民营经济坚持放开领域、放宽条件、放手发展，努力做大做强骨干企业，引导中小企业向“专、精、特、新、强”方向发展。扎实做好市、县政府和乡镇机构改革工作。按照省政府的安排部署，搞好莒县省管县财政体制改革试点。深化农村综合改革，扩大村级公益事业一事一议财政奖补试点，推进集体林权制度改革，稳妥发展多种形式的土地规模经营。

　　(六)进一步提高城乡规划建设管理水平。坚持突出重点、打造亮点，扩展新城区、改造老城区、保护生态区、突出接合部、构筑大框架，着力完善城市功能，优化空间布局，提高城市品位。搞好城市北部地区、涛雒新区、潮河两城河山片区分区规划和城乡一体化发展规划、村居布点规划编制。加强规划批后监管，维护规划的权威性和严肃性。高起点、高标准完成城市中心区规划设计并启动建设，着力将其打造成能够满足国际商务、旅游、休闲、体育等需求，并体现日照特色的高档中央商务区和富有特色的活力街区。加快推进阳光海岸带整治工程，力争5月份完成南段整治任务，尽快启动中段和北段整治，使其成为自然、生态、富有个性的黄金海岸带。以打造水上嘉年华为目标，进一步完善奥林匹克水上公园功能，完成游泳馆建设，搞好城市规划展览馆建设，进一步提升水上运动之都内涵。按照规划先行、合村并居、打造社区、村企合作、综合开发、政府引导、市场化运作的思路与模式，加快城中村改造步伐。

　　扎实做好援建北川县和桃龙乡工作，为“再造一个新北川”作出新贡献。

　　深入推进城乡环境综合整治。突出城乡接合部、城市出入口、旧住宅区、小街小巷及老城区等重点部位，完善供水、排水、供热、燃气管网，建成大学城污水处理站、再生水回用二期工程，开工建设第三污水处理厂。抓好东关北路、北海路、香店河等道路、河道整治建设工程，提升老城区面貌。全面落实生态补偿机制，加大源头治理力度，严格保护海岸带、饮用水源地、山体、河道、森林等重点区域，加快城区与工业园区之间生态隔离带建设。坚持“森林式”与“花园式”相结合，提升城市绿化水平，年内实施4条新建道路绿化、48条现有道路绿化提升和20处绿地景观完善，新增城市绿地面积146万平方米。牢固树立人性化、规范化、法制化的理念，动员社会力量参与城市管理，不断提升城管执法水平。

　　(七)积极构建民生和公共安全体系。坚持把保障和改善民生作为经济社会发展的出发点和落脚点，扎扎实实为群众办实事、做好事、解难事。

　　一是千方百计做好就业工作。认真组织实施“三支一扶”计划，积极引导高校毕业生到农村基层、中小企业和非公有制企业就业，鼓励扶持自主创业。启动政府购买公益岗位计划，年内开发公益性岗位400个，重点用于安置“4050”人员、低保失业人员、贫困残疾人、城乡“双零家庭”、特困家庭高校毕业生等困难群体就业。实施就业创业培训五年规划，搞好军转安置，加大对农村劳动力、就业困难群体、下岗失业人员、未就业高校毕业生的培训和扶持。年内新增城镇就业3万人，实现下岗失业人员再就业8000人，有组织转移农村劳动力1.6万人，城镇登记失业率控制在3.5%以内。

　　二是坚持优先发展教育事业。实施镇域和城区教育均衡发展工程，促进教育公平。深化课程、考试评价制度和招生制度改革，全面推进素质教育。强化各级政府发展普通高中教育的责任，年内将改制高中全部恢复公办性质。加大投入力度，改善高中办学条件。加强职业教育基础能力建设，大力实施校企合作，进一步扩大办学规模，增强为经济建设服务功能。完善教师补充和流动机制，实施绩效工资制度，调动教师工作积极性，提升教书育人能力。认真实施城乡中小学校舍安全工程，推进中小学标准化建设，把学校建成最安全、家长最放心的地方。

　　三是推动医药卫生事业改革发展。搞好医药卫生体制改革，出台配套政策，加强城镇职工和居民医疗保险制度建设，城镇医保覆盖率稳定在90%以上，“新农合”财政补助标准提高到100元。建立健全基层医疗卫生服务体系，加大城乡医疗基础设施投入，抓好市人民医院、市中医院病房楼改扩建工程，完成8处乡镇卫生院建设改造任务，对413处村卫生室业务用房进行整修改造，为397处村卫生室配备基本医疗设备。进一步做好甲型H1N1流感和手足口病等重点传染病的防治。

　　四是健全城乡社会保障体系。加强农村社会保障制度建设，稳妥推进“新农保”试点。深入开展社会保险扩面征缴，积极引导新增就业人员、非公有制经济从业人员、农民工和灵活就业人员参保。进一步提高城乡低保、五保供养和企业退休人员基本养老金标准。在试点的基础上研究出台城乡困难群众医疗救助办法。年内完成市社会福利中心建设任务。完善城市低收入家庭住房保障体系，为750户低收入住房困难家庭发放经济适用房补助，为780户低收入住房困难家庭发放廉租房补贴。

　　五是深入推进公共安全体系建设。探索建立公共安全预防、管理、运行、保障四大机制，提高应对自然灾害、事故灾难、公共卫生和社会安全等突发事件的能力，做好极端气候变化等应急预防工作。扎实开展“安全生产基层基础年”活动，严格落实安全生产责任制，防止重特大安全事故发生。加强农产品、食品、药品安全监管，加快建设市、区县食品药品质量检验检测中心和药品不良反应监测中心，进一步健全从农田到餐桌、从工厂到货架的“链条式”监管模式。正确处理新形势下的人民内部矛盾，健全完善“三全”维稳体系，注重从源头上解决影响社会稳定的问题。加强社会治安综合治理，完善提升“五网”防控及“合成化”现代警务体系，保持对各类违法犯罪活动的严打高压态势，增强人民群众的安全感。

　　(八)大力加强精神文明建设、民主法制建设、社会事业和双拥共建等工作。扎实开展“迎水运、讲文明、树新风”活动，推动“四心”教育向“农村、社区、企业、机关、服务窗口”五个层次延伸。全面加强滨海文化名城建设，大力繁荣发展文化事业和文化产业，积极推进市级综合文化中心、创意产业园等重点项目建设，加快普及乡镇综合文化站、农村文化大院和农家书屋。稳妥推进数字电视整体转换，加快实现有线电视户户通。大力开展全民健身运动，积极备战22届省运会，精心筹办2010年中国水上运动会。全面落实计划生育优先优惠政策，加强计生阵地和队伍网络建设，千方百计稳定低生育水平。广泛动员全社会的力量，扎实做好第六次人口普查工作。认真落实“五五”普法规划，积极开展法治创建和法律服务。依法搞好村委会换届选举工作。积极做好双拥共建和优抚安置工作，争创全国双拥模范城“四连冠”。加强民兵预备役建设，提高国防动员质量。

　　各位代表：

　　明年将进入第十二个五年规划时期。“十二五”是我市深入贯彻落实科学发展观、全面建设小康社会的关键阶段，也是加快打造蓝色经济区、建设海洋特色新兴城市的重要时期。我们将组织力量精心编制“十二五”发展规划，坚持有所为有所不为，突出重点领域和重大项目，特别是把培育产业集群作为规划编制的重点，加强与国家、省规划的衔接，争取更多的项目进入国家、省的盘子。解放思想、锐意创新，集思广益、汇集民智，确保编制出一个符合日照实际的发展规划，为推动全市科学发展、跨越发展、和谐发展提供科学依据和行动指南。要通过这项工作，努力以全市广大干部群众的聪明才智展现日照发展的宏伟蓝图！

　　三、切实加强政府自身建设

　　新的形势和任务对政府自身建设提出了新的要求。我们将认真贯彻落实党的十七届四中全会及中央和省的一系列重要精神，按照人民群众对政府工作的期待和愿望，有针对性地改进和加强我们的工作，进一步坚持“为民、务实、清廉”，努力建设服务型政府。

　　一是积极为群众排忧解难。政府各级领导干部要按照建设服务型政府的要求切实转变职能、改进作风，扎扎实实地为基层和群众办实事、解难题。进一步拓宽民意诉求渠道，充分发挥市长公开电话、市长信箱、短信平台、网络对话的作用，及时处理群众反映的困难和问题。把更多的财政资金用于解决民生问题，把更多的公共资源投向农村、基层和困难群众，特别是高度关注下岗职工、老弱病残、鳏寡孤独、未就业毕业生及城乡低保对象等困难群体，及时帮助他们解决实际问题，让广大群众共享改革发展成果。精心组织实施2010年度为民办实事重点项目，确保按时完成。根据政策调整变化和群众需要，继续搞好《民生手册》的增编发放工作。

　　二是深入推进政府工作提速。结合开展政府机构改革进一步转变职能，深入推进政企、政资、政事、政府和中介组织“四分开”，发挥市场在资源配置中的基础性作用。继续深化行政审批制度改革，采取取消、归并、调整等措施，大幅度精简审批事项，使总体审批事项继续少于周边城市。按照规范、高效、便民、廉洁的要求，在各级行政服务大厅实行行政审批职能和审批项目“两集中”、行政审批人员和权限“两到位”，减少审批环节，提高行政效能。严格执行行政问责制和绩效管理制，强化政府工作人员的执行意识、落实意识和责任意识，力求杜绝“生、冷、硬、横、顶、拖”等衙门作风，对“不作为、慢作为、乱作为”行为，发现一起，查处一起，决不姑息迁就。

　　三是坚持依法行政和民主决策。各级政府都要按照法定权限和程序行使权力、履行职责，把依法行政贯穿于决策、执行、监督全过程。继续坚持政府常务会议集体学法制度，不断增强依法行政意识。健全完善行政执法评议考核机制，加强行政执法监督检查，促进规范执法。完善重大问题集体决策、专家论证、社会听证和合法性审查等制度，提高政府决策水平。自觉接受人大及其常委会的法律监督和政协的民主监督，广泛听取民主党派、工商联、无党派人士和各人民团体的建议意见，主动接受社会舆论和人民群众的监督。强化监察、审计专门监督，做到行政权力运行到哪里、监督就落实到哪里，财政资金运用到哪里、审计就跟进到哪里。坚持“公开是原则，不公开是例外”，深入推进政府信息公开，打造“阳光政府”。

　　四是切实加强廉政建设。坚持廉洁从政、从严治政，加快推进惩治和预防腐败体系建设，严格规范规划、用地、工程招投标、财政资金使用、政府采购、国有资产转让、工程质量监理等公共资源管理权力运行，大力推进公共资源交易市场化改革，建立预防腐败的长效机制。积极采用现代科技手段预防腐败，加快建设覆盖市和区县所有行政部门的综合电子监察系统。牢固树立艰苦奋斗过“紧日子”的思想，扎实推进节约型机关建设，严格控制车辆、会议、庆典、出国出境等支出，坚决反对铺张浪费。各级领导干部要认真落实党风廉政建设责任制，严格履行“一岗双责”，切实把好自身、分管部门、家人与身边工作人员“三个关口”，保持清正廉洁，一身正气。

　　各位代表：

　　日照的发展已经进入了一个崭新的时期。时代赋予重托，奋斗铸就辉煌。我们坚信，任何挑战都阻挡不住日照实现跨越发展的坚定步伐，任何困难都动摇不了全市人民创造美好生活的坚定信念。让我们紧密团结在以胡锦涛同志为总书记的党中央周围，在中共日照市委的坚强领导下，坚定信心，振奋精神，抢抓机遇，加快发展，为全面实现“十一五”时期经济社会发展目标，夺取全面建设小康社会新胜利，加快建设海洋特色新兴城市而努力奋斗！
